# Biochemical, morphological and molecular assessments of *n* butanol fraction of *Phoenix dactylifera L*. following exposure to inorganic mercury on the liver of Wistar rats

**DOI:** 10.1186/s42826-024-00203-9

**Published:** 2024-04-19

**Authors:** Musa Garba Abubakar, AN Agbon, SA Musa, WO Hamman, SB Oladele

**Affiliations:** 1 Microscopy and Stereology Research Unit, Department of Human Anatomy, Ahmadu Bello University, Zaria, Nigeria; 2 Nigerian Defence Academy, Kaduna, Nigeria; 3 Department of Veterinary Pathology, Faculty of Medical Sciences, Ahmadu Bello University, Zaria, Nigeria

**Keywords:** Histochemical, Immunohistochemical, Silymarin, Oxidative stress, Hepatotoxicity

## Abstract

**Background:**

Mercury chloride (HgCl_2_) damages tissues it comes in contact with in sufficient concentration. This study evaluated the protective effects of *n-*butanol fraction of *Phoenix dactylifera* (BFPD) on mercury-triggered liver toxicity in Wistar rats. 25 male rats were divided into 5 groups of 5 rats each. Group I was administered 2 ml/kg of distilled water; group II was administered 5 mg/kg of HgCl_2_; group III was administered 500 mg/kg of BFPD + 5 mg/kg of HgCl_2_; group IV was administered 1000 mg/kg of BFPD + 5 mg/kg of HgCl_2_, while group V was administered 100 mg/kg of silymarin + 5 mg/kg of HgCl_2_. orally for 2 weeks. The rats were euthanized and liver tissue blood samples were collected for histological, histochemical, stereological, immunohistochemical, molecular, and biochemical studies.

**Results:**

The results revealed that HgCl_2_ induced oxidative stress in the rats evident by histoarchitectural distortions and altered levels of liver enzymes, proteins, and oxidative stress biomarkers when compared to the control. However, BFPD treatment restored these changes. Glutathione peroxidase levels decreased (*p* < 0.05) in the HgCl_2−_treated group when compared to the control and BFPD-treated groups. HgCl_2_ group revealed reduced reactivity with histochemical and immunohistochemical stains (Masson’s Trichrome and B cell Lymphoma 2) when compared to the control, with a significant decrease in quantified liver Bcl-2 stain intensity when compared to the silymarin-treated group. BFPD administration revealed normal staining intensity comparable to the control. HgCl_2_ administration revealed a remarked decrease in the number of hepatocytes when compared to the control, BFPD, and silymarin groups. BFPD preserved (*p* < 0.05) the stereological features when compared to the HgCl_2_-treated group. GPx activity in the liver decreased (*p* < 0.05) with HgCl_2_ administration when compared to the control and silymarin-treated groups. BFPD attenuated GPx gene activity to levels similar to the control indicating some level of amelioration against HgCl_2_-induced toxicity.

**Conclusions:**

The ability of BFPD to mitigate HgCl_2_ triggered liver alterations could be attributed to the antioxidant property of its flavonoid content. Therefore, BFPD may be a potential candidate for treating and managing liver-induced mercury intoxication.

## Background

Mercury is a heavy metal and an environmental and industrial pollutant that induces severe alterations in body tissues such as the liver [1, 2]. Mercury poisoning can result from inhalation, ingestion, or absorption through the skin [3]. It exerts deleterious effects on biological systems by generating reactive oxygen species (ROS) which results in disruption of vital cellular metabolic processes [4]. The liver suffers significantly from mercuric chloride poisoning through oxidative stress [5]. Massive efforts are being made to discover new drugs that could counteract mercurial toxicity, as the use of chelating agents presents adverse side effects [6]. Hence, a need to evaluate plants for their medicinal properties.

Date palm (*Phoenix dactylifera* L.) fruits are widely used in traditional medicine for the treatment of various disorders, e.g., fever, inflammation, and oxidative stress [7]. It is also used to treat liver diseases [8]. *Phoenix dactylifera* has been demonstrated to have hepato-protective, anti-inflammatory, and antioxidant activity [9, 10]. Hence, the need to evaluate the potential of *Phoenix dactylifera* as a hepatoprotective agent following exposure to mercury chloride.

This study evaluated the hepatoprotective effect of *n*-butanol fraction of *Phoenix dactylifera* against mercury chloride-triggered hepatotoxicity in Wistar rats.

## Methods

### Experimental animals

A total of twenty-five male Wistar rats weighing (145 ± 15 g) were obtained from the Animal Facility of Pharmacology, Ahmadu Bello University (ABU), Zaria. Rats were kept under standard laboratory conditions in the Animal Facility of the Department of Human Anatomy, ABU, Zaria, where they were acclimatized for fourteen days before the commencement of the experiments. The rats were fed with rat chow and water was allowed *ad libitum*. The treatment groups were administered a combination of either mercury + *n*- butanol fraction of *P. dactylifera* or mercury + silymarin. Rats were weighed at the beginning and the end of the study.

### Plant material

Dried fruits of *Phoenix dactylifera* L. (date palm) were obtained and authenticated in the Herbarium Unit, Department of Biological Sciences, ABU, Zaria, and a Voucher Number provided: 7130.

### Chemical and drugs

Mercury: 50 g of mercuric chloride, a whitish powdered substance, was obtained from a reputable Chemical Store, and used as a hepatotoxicant. The product was manufactured by British Drug Houses (BDH) Chemicals, Poole, England.

### Chloroform

Chloroform: 500 ml chloroform (≥ 99.8%), a colorless and volatile solvent, was obtained and used as an anesthetic agent. The product was manufactured by British Drug Houses (BDH) Chemicals, Poole, England.

### Silymarin

Silymarin: Silymarin (Silybon‑70®; tablets 70 mg) was obtained from a Pharmaceutical Store and used as standard (reference) antioxidant drug. The product was manufactured by Micro Labs Limited, India.

### Plant extraction and fractionation

Preparation of *n*-butanol fraction of *Phoenix dactylifera* fruit pulp (BFPD) was conducted in stages involving different solvents of extraction: methanol, ethyl acetate and *n*-butanol according to the method described by Kriaa [11]. The preparation was carried out in the Department of Pharmacognosy and Drug Development, ABU, Zaria.

### Experimental design

**Fig. 1:**
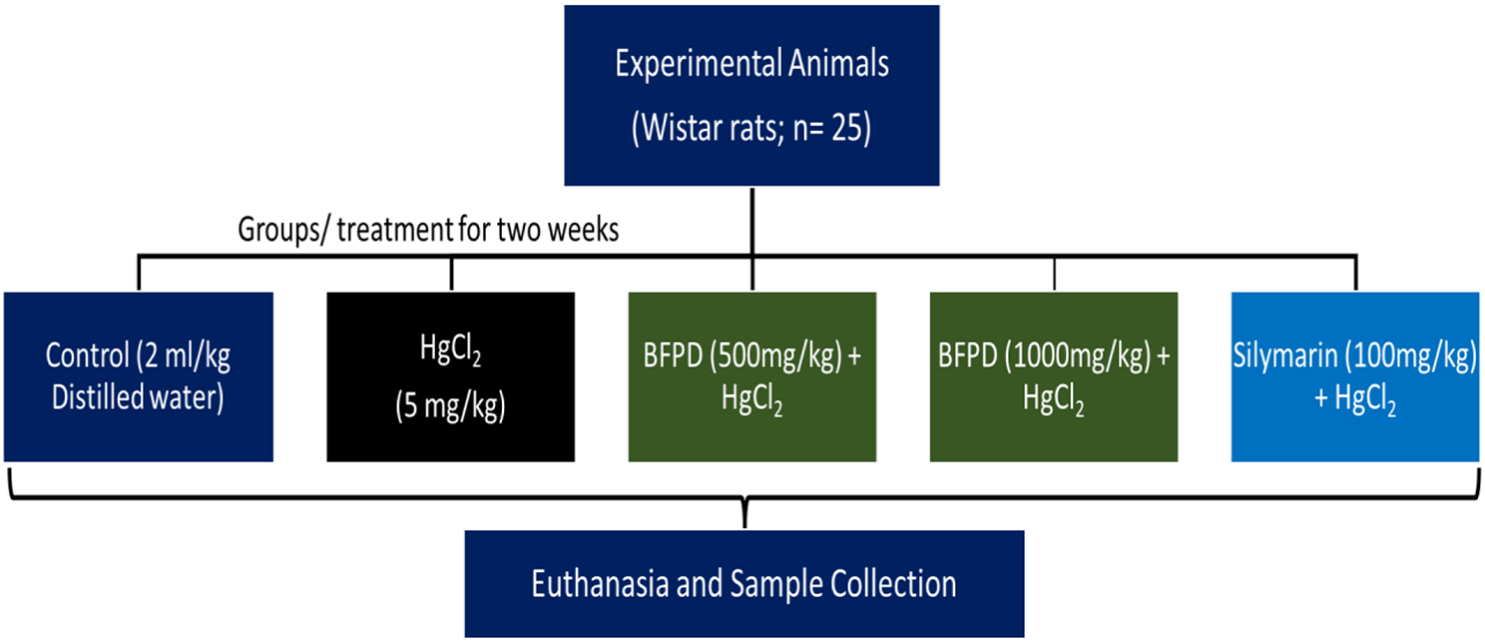
Experimental design. All administrations were via oral route. Mercuric chloride (HgCl_2_); n-butanol fraction of Phoenix dactylifera fruit pulp (BFPD)

Twenty-five rats were divided into five categories (Categories I– V; *n* = 5). Category I served as the control and was administered 2 ml/kg distilled water. Category II was administered mercuric chloride (HgCl_2_) 5 mg/kg (12.5% LD_50_ as reported by Sheikh [12]). Categories III and IV were administered 500 mg/kg and 1000 mg/kg BFPD followed by 5 mg/kg HgCl_2,_ respectively. Category V was administered 100 mg/kg Silymarin (as reported by Issa [13]) followed by 5 mg/kg HgCl_2_. The administrations were via oral route and lasted for a period of fourteen days (Fig. 1).

### Animal sacrifice

At the end of the experiment, the rats were euthanized under chloroform anesthesia, The thoracic cavity was dissected and blood sample collected via cardiac puncture into plain sample bottles for biochemical assessments. The abdominal cavity was equally dissected and the liver was harvested for histochemical, stereological and molecular studies.

### Body and organ weights

Before euthanasia absolute body weights of the animals were measured at the beginning (Initial Weight) and, at the end (Final Weight) of the experiment. Percentage weight change was computed as described by Agbon et al. [14], and means were compared between the groups. The liver was also weighed. Relative organ weight (Organosomatic index) was computed according to the method of Rahardjo et al. [15], as described by Agbon et al. [14] [(organ weight/ final body weight) ×100] and values obtained were analyzed and compared between the groups.

### Histological and histochemical studies

Harvested organ (liver) was fixed in 10% buffered formal saline and processed using histological techniques for light microscopic examination. Processed histological sections were stained with Hematoxylin and Eosin (H&E) to demonstrate general histoarchitectural features. Sections were equally stained using histochemical stain (Periodic Acid Schiff (PAS) and Masson’s Trichrome (MT)) to demonstrate glycogen moiety and reticulin fibers, respectively. Histological and histochemical tissue processing was carried out in the Histology Unit of the Department of Human Anatomy, ABU, Zaria.

### Immunohistochemical studies

Immunohistochemical techniques was adopted to demonstrate apoptotic changes in hepatocytes using the immuno-staining, B Cell Lymphoma 2 (Bcl-2) as described by Caglayan [16]. The processed liver tissue sections were stained with Bcl-2 antibody according to the manufacturer’s instructions for light microscopic examination. Immunohistochemical technique was carried out in the Department of Chemical Pathology, ABU Teaching Hospital Shika, Zaria.

### Image analysis - quantification of PAS and Bcl-2 reactivity

PAS and Bcl-2-stained micrographs were quantified for staining reactivity by adopting the method described by Amber [17], which involved measuring staining intensity using a computer running image analysis software (ImageJ® NIH, US) according to the manufacturer’s specifications. The ImageJ® region of interest (ROI) manager tool for analysis of specific areas of the micrographs was employed. The mean gray values for three ROI were obtained, means were computed and analyzed.

### Stereological studies

Stereological analysis was conducted to estimate the number of hepatocytes in rats. The paraffin-processed liver samples were serially sectioned as reported by Gundersen and Jensen [18], to provide sections of 10ʯm thickness. 25 tissue sections were selected per group using a systematic uniform random sampling method and stained with H and E. An unbiased estimate of the number of normal hepatocytes was obtained using the physical fractionator method as reported by Yurt [19].

### Molecular studies

A piece from the harvested liver was excised, carefully wrapped in sanitized foil paper, preserved in dry ice packs and transported to a DNA laboratory for molecular assessment. Sample preparation involved the following: Excised samples were mechanically homogenized, centrifuged and aliquots of the supernatant were obtained for molecular analysis. GPx gene expression (forward; 5’-CCTGGTATCTGGGCTTGGTG-3’ reverse; 5’-TTAGGCGTAAAGGCATCGGG-3’ [20], was assayed using Real-Time Polymerase Chain Reaction (RT-PCR). β-actin gene (forward; 5’-GGCATCCTGACCCTGAAGTA-3’ reverse; 5’-GGGGTGTTGAAGGTCTCAAA-3’ [21], was used as the reference housekeeping gene. Molecular assessments were conducted in the DNA Labs, Kaduna, Nigeria.

### Biochemical studies

Biochemical assessments were conducted using the serum of blood samples collected to assay for liver function - Alanine transaminase (ALT), alkaline phosphatase (ALP), and aspartate aminotransferase (AST); liver serum proteins: Albumin (AB), Globulin (GB) and total proteins (TP); oxidative stress biomarkers and antioxidant enzyme activity: malondialdehyde (MDA), Superoxide dismutase (SOD), Catalase (CAT) and Glutathione peroxidase (GPx). Biochemical assessments were conducted in the Department of Chemical Pathology, Faculty of Basic Clinical Sciences, Ahmadu Bello University Teaching Hospital, Shika.

### Data analysis

Results obtained were analyzed using GraphPad Prism (*version 9.3*) and results were expressed as mean ± S.E.M. The presence of significant differences among means of the groups were determined using one-way analysis of variance (ANOVA) with *Tukey post hoc test*. Paired sample *t–test* was employed for the comparisons of means as appropriate. Values were considered significant when *p* < 0.05.

## Results

Qualitative phytochemical screening of n-butanol fraction of Phoenix dactylifera fruit pulp indicating the presence of secondary metabolites was carried out (Table 1). A yield of 12.44% (62.2 g) was obtained for n-butanol fraction.

**Table 1:** Phytochemical constituents of *n*-butanol fraction of *Phoenix dactylifera*

Constituents	Inference
Alkaloid	-
Anthraquinone	-
Cardiac glycoside	+
Flavonoid	+
Saponin	+
Steroid and tripenone	+
Tannins	+

+ = Present

- = Absent

The table above shows the result of the qualitative phytochemical screening of *n* butanol fraction of Phoenix dactylifera. The negative sign indicates the absence of a phytochemical constituents while the positive sign indicates the presence of the phytochemical.

### Physical observation result

The physical activities of experimental animals including agility and behavioral patterns especially feeding habits were observed during the period of administration. Wistar rats in the control group were observed to exhibit normal movement and playfulness, whereas rats in the treatment groups exhibited reduced activities such as sluggishness and loss of appetite especially in the mercury (HgCl_2_) treated group.

### Body weight and organosomatic index results

**Fig. 2:**
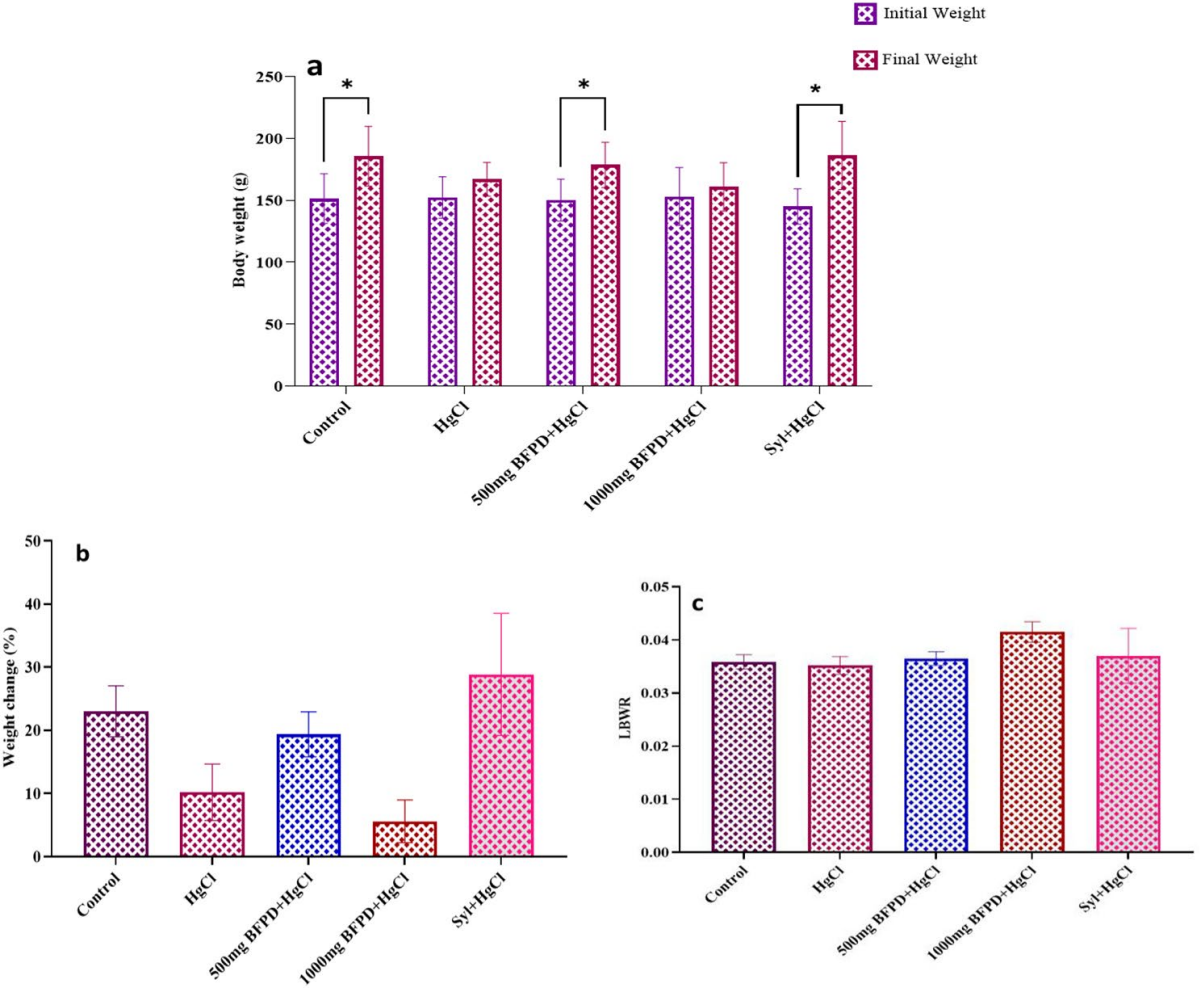
**(a)** Comparison of absolute body weight of Wistar rats. *n* = 5, mean ± SEM, Paired sample t-test, *=*p* < 0.05 when compared initial and final weight of the groups. HgCl_2_ = Mercury chloride (5 mg/kg), BFPD = *n*-Butanol fraction of *Phoenix dactylifera* (500 mg/kg; 1000 mg/kg), Syl = silymarin (100 mg/kg). **(b)** Effect of BFPD on percentage (%) weight change of Wistar rats. *n* = 5, mean ± SEM, one-way ANOVA, *p* < 0.05 when compared across the group. HgCl_2_ = Mercury chloride (5 mg/kg), BFPD = *n*-Butanol fraction of *Phoenix dactylifera* (500 mg/kg; 1000 mg/kg), Syl = silymarin (100 mg/kg). **(c)** Effect of BFPD on Liver body weight ratio of Wistar rats. *n* = 5, mean ± SEM, one-way ANOVA, *p* < 0.05 when compared across the group. HgCl_2_ = Mercury chloride (5 mg/kg), BFPD = *n*-Butanol fraction of *Phoenix dactylifera* (500 mg/kg; 1000 mg/kg), Syl = silymarin (100 mg/kg)

The absolute body weights of the Wistar rats were measured and the initial and final weights were compared. The result showed that weight increase was observed in all treated groups with a remarkable increase in the control, BFPD, and silymarin-treated groups (Fig. 2a). Weight change assessment revealed no significant difference in all treated groups when compared across all groups (Fig. 2b).

The organosomatic index (relative organ weight) of the liver revealed no significant difference in all treated groups when compared across all groups. (Fig. 2c).

### Histological and histochemical assessments

**Fig. 3:**
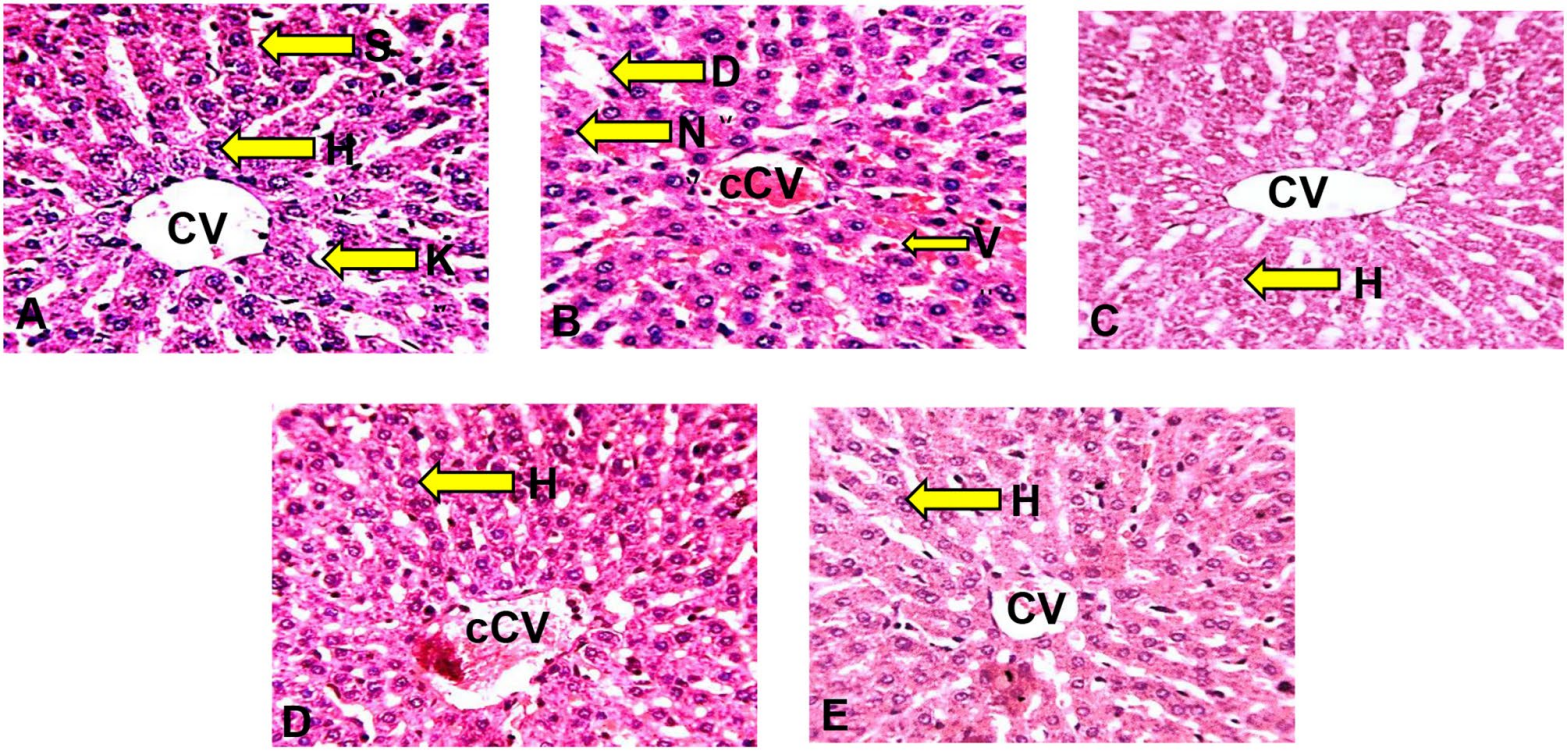
Section of the liver of Wistar rats (H & E x 250) **(A)**: Photomicrograph of the transverse section of liver of Wistar rats administered 2 ml/kg H_2_0 (control group) with normal histoarchitectural features. H&E ×250. CV: Central vein; K: Kupffer cell; H: Hepatocytes; S: Sinusoids. **(B)**: Photomicrograph of the transverse section of liver of Wistar rats administered HgCl_2_ with distorted histoarchitectural features. H&E ×250. cCV: Congested central vein; N: Pyknotic nuclei; D: Sinusoidal dilatation; V: Hepatocellular vacuolation. **(C)**: Photomicrograph of the transverse section of liver of Wistar rats administered 500 mg/kg BFPD and HgCl_2_ with normal histoarchitectural distortion. H&E ×250. CV: Central vein; H: Hepatocytes. **(D)**: Photomicrograph of the transverse section of liver of Wistar rats administered 1000 mg/kg BFPD and HgCl_2_ with mild distorted histoarchitectural features. H&E ×250. cCV: Congested central vein; H: Hepatocytes. **(E)**: Photomicrograph of the transverse section of liver of Wistar rats administered with silymarin and HgCl_2_ showing relatively normal histoarchitectural features. H&E ×250. H: Hepatocytes; CV: Central vein

Histological sections of Wistar rats’ liver stained with Hematoxylin and Eosin (H&E) to demonstrate general histological features, Periodic Acid Schiff, (PAS) to demonstrate glycogen moiety and Mason’s Trichome to demonstrate reticular fibers, and examined under the light microscope revealed the following:

The examination of the liver sections of rats in the control group revealed normal histoarchitectural features of the liver parenchyma observed by the characteristic appearance of the hepatic lobule unit, centrilobular venules (central veins), array of anastomosing plates of hepatocytes radiating from the central vein separated by sinusoids (vascular spaces) and portal triad (hepatic portal vein, hepatic artery, and bile duct) (Fig. 3A).

The HgCl_2_–treated group revealed distortions of the histoarchitectural features of the liver as hepatocellular vacuolation, congestion of the central vein, sinusoidal dilatation, and pyknotic nuclei when compared to the control (Fig. 3B). The *n*-butanol fraction of *P*. *dactylifera* (BFPD) + HgCl_2_ treated groups revealed mild distortion of the liver histoarchitecture evident as congestion of the central vein when compared to the control (Fig. 3C and D). Silymarin + HgCl_2_ treated group showed preserved histoarchitectural features of the liver when compared to the control (Fig. 3E).

**Fig. 4:**
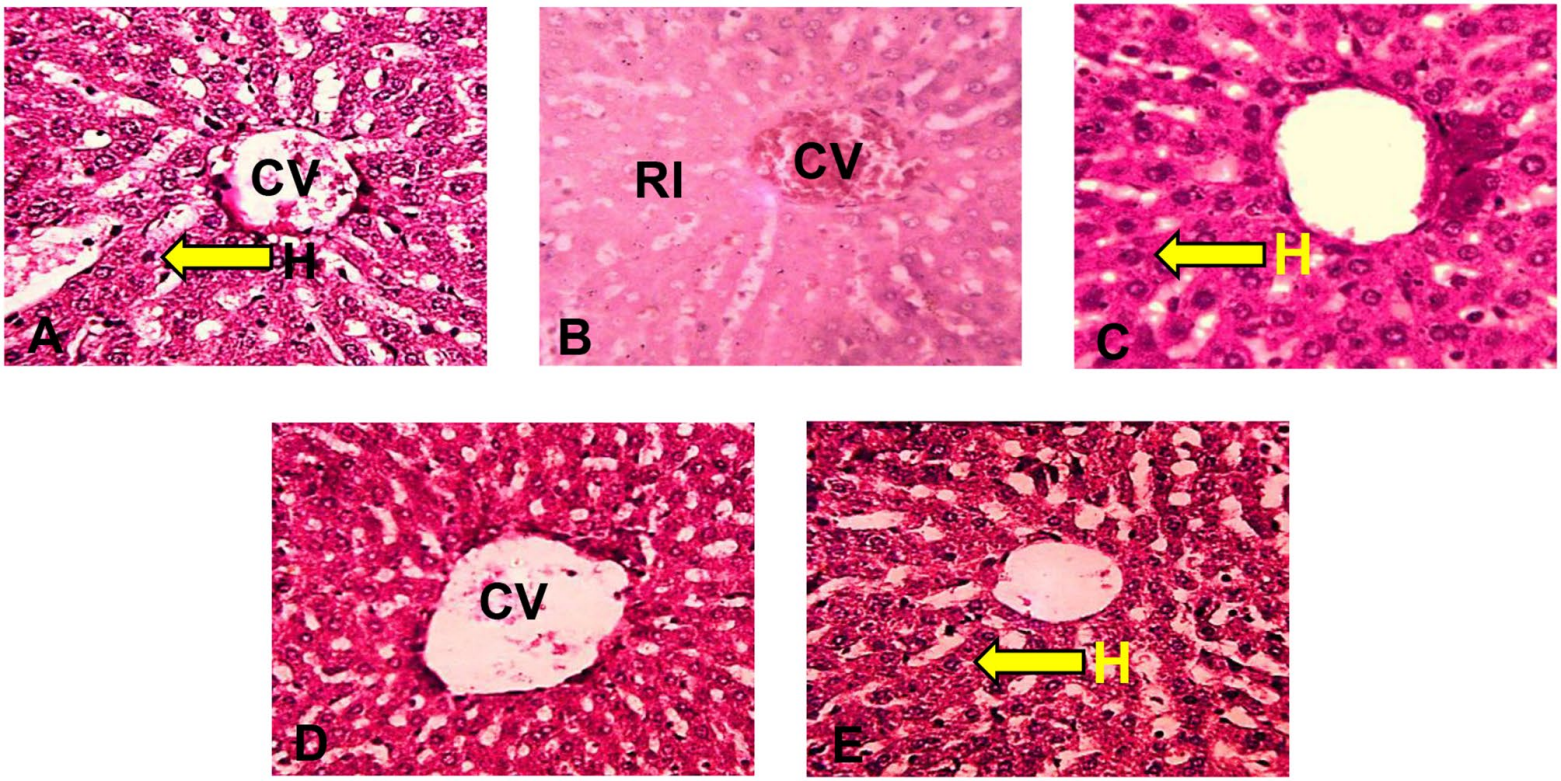
Sections of the liver of Wistar rats (PAS x250). **(A)**: Photomicrograph of the transverse section of liver of Wistar rats administered 2 ml/kg H_2_0 (control group) with normal staining intensity. H: Hepatocytes; CV: Central vein. **(B)**: Photomicrograph of the transverse section of liver of Wistar rats administered HgCl_2_ with reduced staining intensity. CV: Central vein; RI: Reduced Stain Intensity. **(C)**: Photomicrograph of the transverse section of Liver of Wistar rats administered 500 mg/kg BFPD and HgCl_2_ relatively normal staining intensity; CV: Central Vein; H: Hepatocytes. **(D)**: Photomicrograph of the transverse section of Liver of Wistar rats administered 1000 mg/kg BFPD and HgCl_2_ with normal staining intensity. CV: Central Vein. **(E)**: Photomicrograph of the transverse section of Liver of Wistar rats administered Silymarin and HgCl_2_ with relatively normal staining intensity. H: Hepatocytes

Histochemical (PAS) staining for glycogen moiety revealed positive reactivity to PAS demonstrating the presence of membrane-bound and cytoplasmic glycogen moiety in hepatocytes of the control group (Fig. 4A). However, reduced reactivity to PAS was observed in the HgCl_2–_ treated group indicating depletion of glycogen moiety in the cytoplasm of the hepatocytes as compared to the control (Fig. 4B). BFPD + HgCl_2_ and silymarin + HgCl_2_ treated groups revealed PAS-positive stain intensity comparable to the control (Fig. 4C - E).

**Fig. 5:**
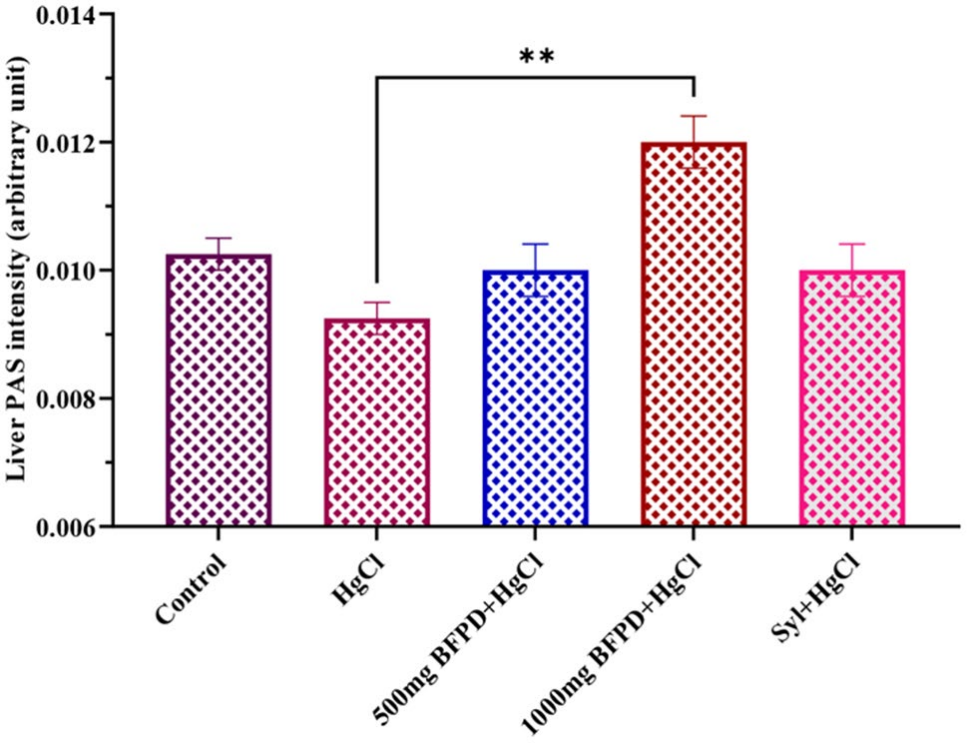
Effect of BFPD on liver PAS intensity of Wistar rats. *n* = 5, mean ± SEM, one way ANOVA, **= *p* < 0.05 when BFPD 1000 mg was compared to HgCl_2_ group. HgCl_2_ = Mercury chloride (5 mg/kg), BFPD = *n*-Butanol fraction of *Phoenix dactylifera* (500 mg/kg; 1000 mg/kg), Syl = silymarin (100 mg/kg)

Quantification of liver PAS reactivity decreased (*p* > 0.05) in the HgCl_2_-treated group in relation to the control. All other treated groups revealed an increase in reactivity with a remarkable increase observed in BFPD (1000 mg/kg) + HgCl_2_ as compared to the HgCl_2_-treated group (Fig. 5).

**Fig. 6:**
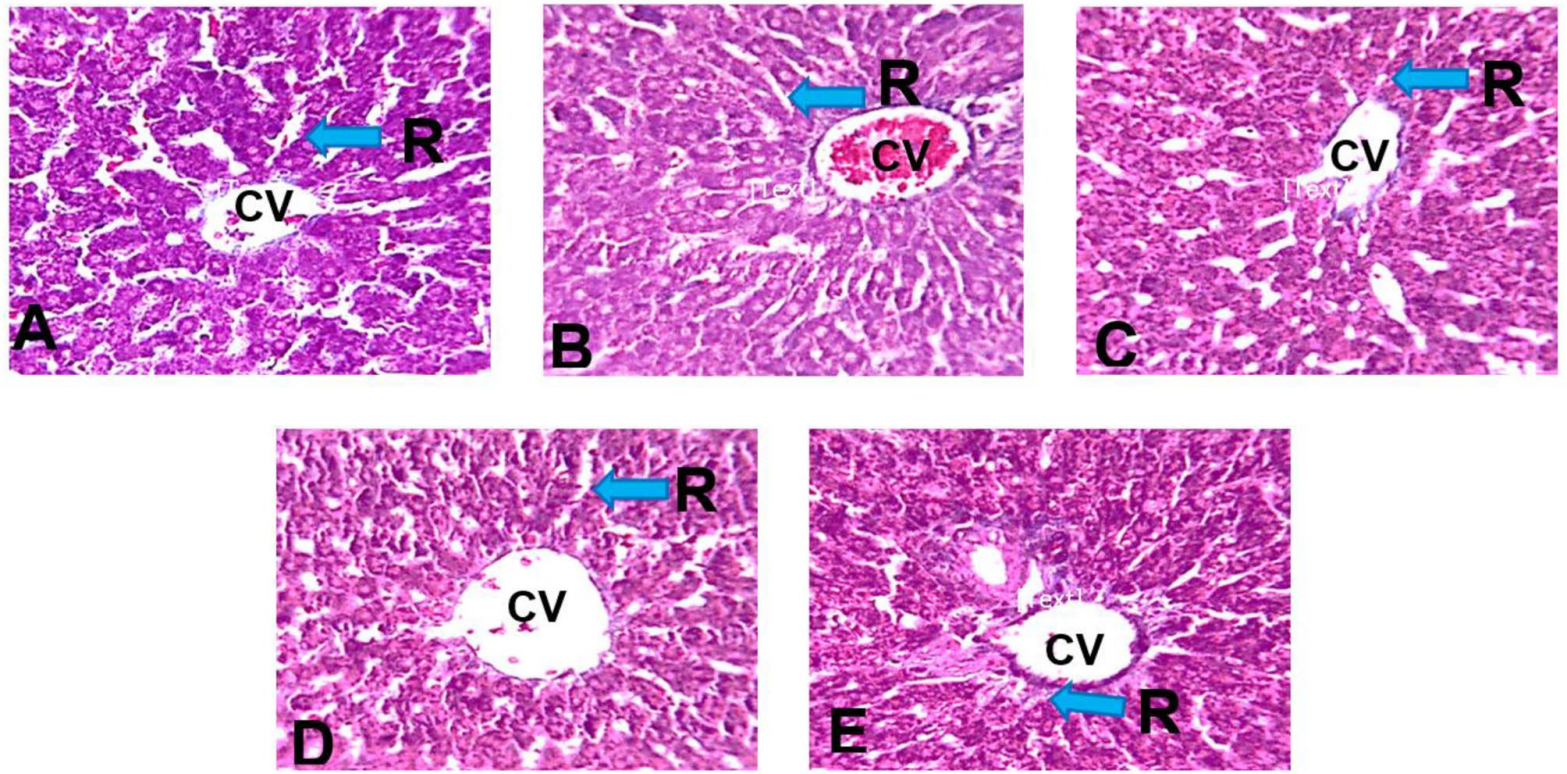
The liver section of Wistar rats (MT x250). Plate **(A)**: Section of liver of Wistar rats administered 2 ml/kg with radiating reticular fibers. CV: Central Vein, R: Radiating reticular fibers. Plate **(B)**: Section of liver of Wistar rats administered HgCl_2_ with radiating reticular fibers. CV: Central Vein, R: Radiating reticular fibers. Plate **(C)**: Section of Liver of Wistar rats 500 mg/kg BFPD and HgCl_2_ showing reticular. CV: congested Central Vein. R: Reticular fibers. Plate **(D)**: Section of liver of Wistar rats administered 1000 mg/kg BFPD HgCl_2_ showing reticular fibers CV: Central Vein, R: Radiating reticular fibers. Plate **(E)**: Section of Liver of Wistar rats administered with silymarin and HgCl_2_ showing radiating reticular fibers. CV: Central Vein, R: Radiating reticular fibers

Histological sections of rats’ liver stained with Mason trichome (MT) revealed normal reticulin framework around the basement membrane of the central veins and periphery of the sinusoids in the control (Fig. 6A). Relative to the control, the treated groups revealed no distinct difference in the reticulin framework. (Fig. 6B - E).

### Immunohistochemical assessments

**Fig. 7:**
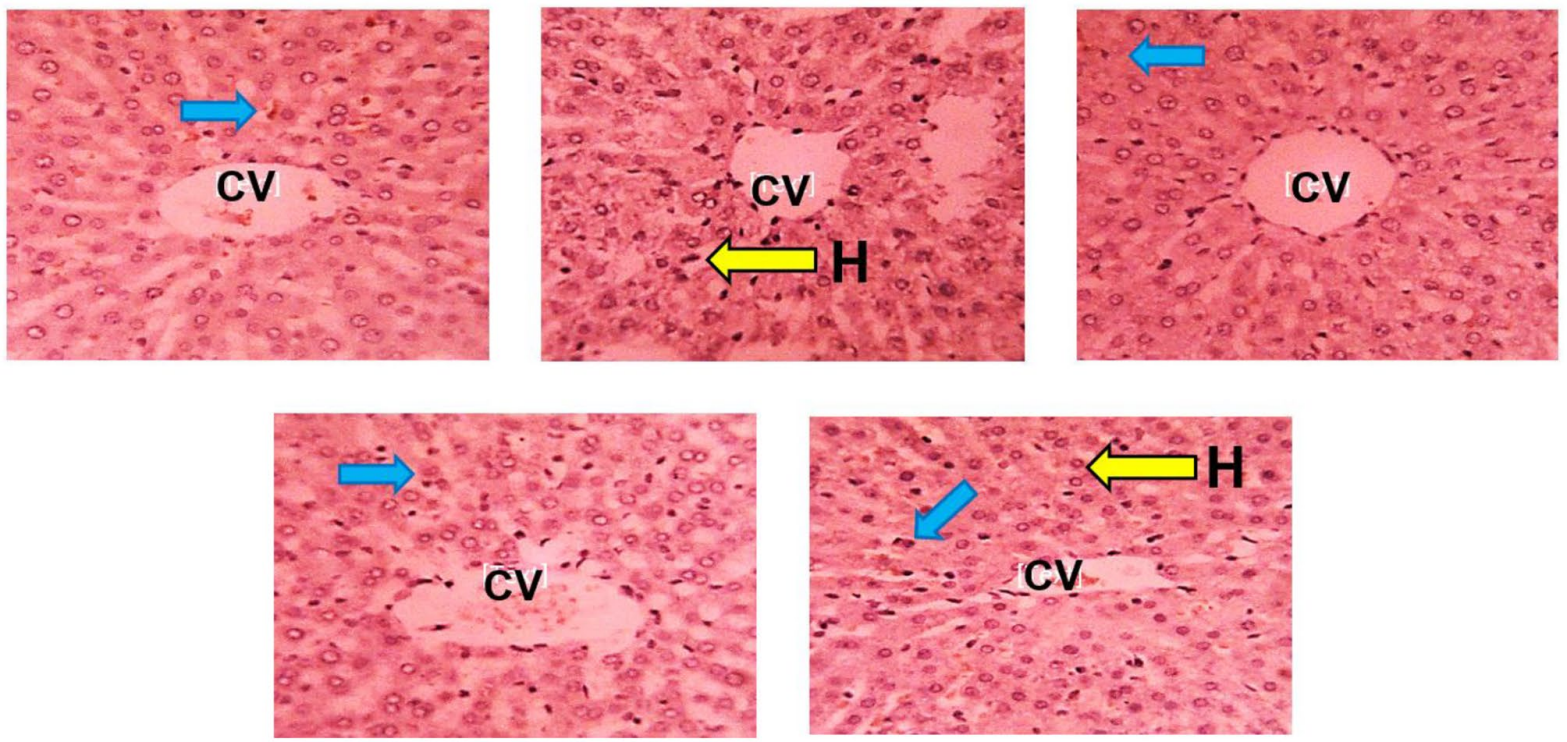
The liver section of Wistar rats (Bcl-2 × 250). Plate **(A)**: Section of liver of Wistar rats administered 2 ml/kg H_2_0 (control group) showing presence of Bcl-2-stained bodies. CV: Central Vein, Blue arrow: Bcl-2-stained cells. Plate **(B)**: Section of liver of Wistar rats administered HgCl_2_ with no Bcl-2-stained bodies. H: Hepatocytes; CV: Central Vein. Yellow arrow: Hepatocytes. Plate **(C)**: Section of Liver of Wistar rats administered 500 mg/kg BFPD and HgCl_2_ with Bcl-2-stained bodies. blue arrow = BCL-2-stained cells. Plate **(D)**: Section of Liver of Wistar rats administered 1000 mg/kg BFPD and HgCl_2_ with few Bcl-2-stained bodies present. CV: Central Vein; blue arrow: Bcl-2-stained cells. Plate **(E)**: Section of Liver of Wistar rats administered silymarin and HgCl_2_ showing numerous Bcl-2-stained bodies. H: Hepatocytes; CV: Central Vein; blue arrow: Bcl-2-stained cells. Yellow arrow: Hepatocytes

Immunohistochemical sections of rats’ liver stained with the immunostain, Bcl-2 revealed positive reactivity with the cells of liver demonstrating antiapoptotic activity in the control group (Fig. 7A). However, the HgCl_2_-treated group revealed reduced reactivity with the cells of the liver when compared to the control (Fig. 7B). BFPD + HgCl_2_ and silymarin + HgCl_2_ treated groups revealed preserved immunoreactivity with the hepatocytes comparable to the control (Fig. 7C - E).

**Fig. 8:**
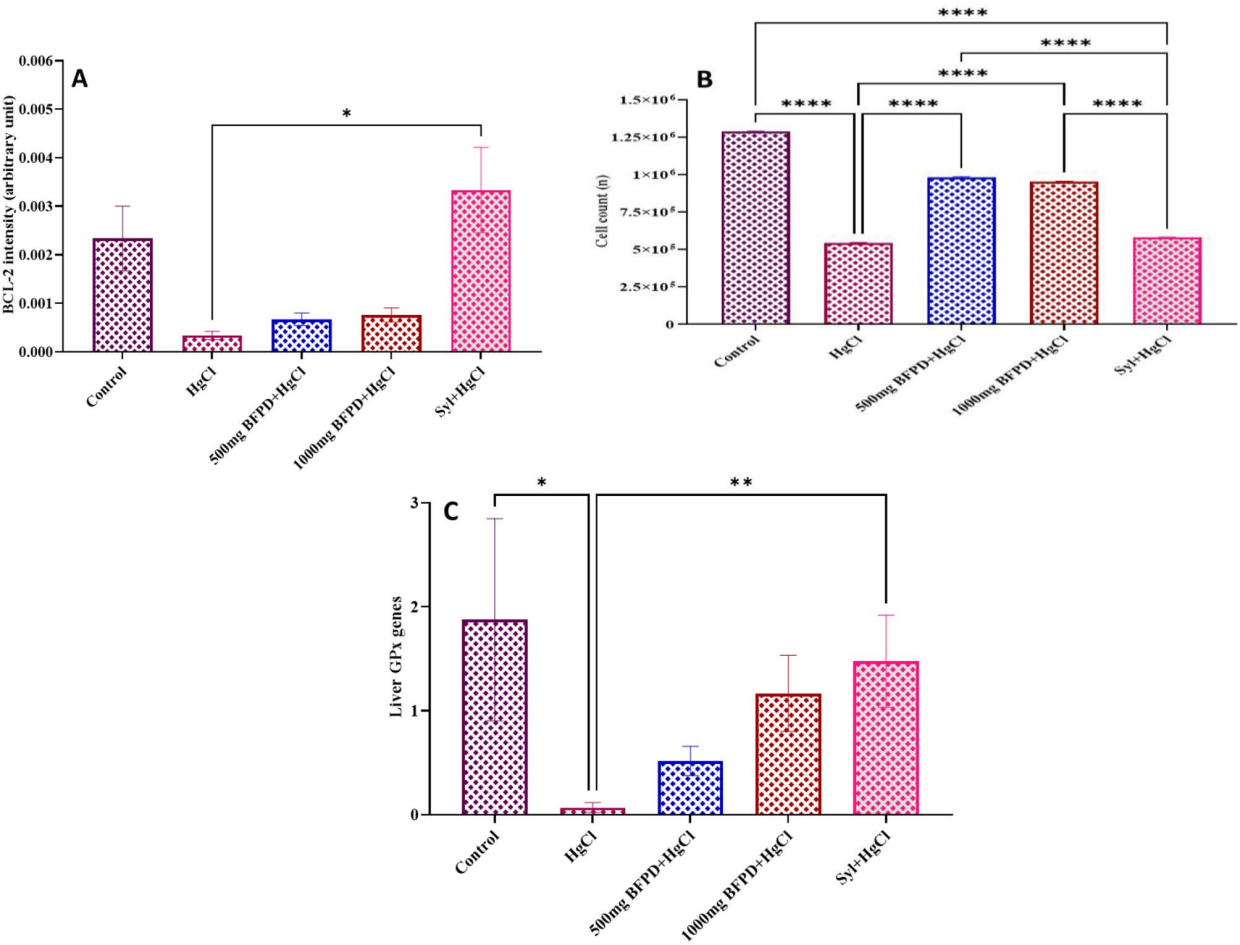
**(A)** Effect of BFPD on liver BCL-2 intensity of Wistar rats. *n* = 5, mean ± SEM, one way ANOVA, **=*p* < 0.05 when HgCl_2_ group was compared to Silymarin group. HgCl_2_ = Mercury chloride (5 mg/kg), BFPD = *n*-Butanol fraction of *Phoenix dactylifera* (500 mg/kg; 1000 mg/kg), Syl = silymarin (100 mg/kg). **(B)** Effect of BFPD on hepatocyte cell count of Wistar rats. *n* = 5, mean ± SEM, one way ANOVA, ****=*p* < 0.05 when compared across the groups. HgCl_2_ **=** Mercury chloride (5 mg/kg), BFPD = *n*-Butanol fraction of *Phoenix dactylifera* (500 mg/kg; 1000 mg/kg), Syl = silymarin (100 mg/kg). **(C)** Effect of BFPD on liver GPx genes expression of Wistar rats. *n* = 5, mean ± SEM, one way ANOVA, *=*p* < 0.05 when control was compared to HgCl_2_ group, and **=*p* < 0.05 when HgCl_2_ group was compared Silymarin group. HgCl_2_ = Mercury chloride (5 mg/kg), BFPD = *n*-Butanol fraction of *Phoenix dactylifera* (500 mg/kg; 1000 mg/kg), Syl = silymarin (100 mg/kg)

Immuno-quantification of liver Bcl-2 reactivity decreased (*p* > 0.05) in all treated groups except the silymarin + HgCl_2_-treated group when compared to the control (Fig. 8A).

### Stereological assessments

Stereological estimation of hepatocytes revealed a decreased (*p* < 0.05) count in the HgCl_2_-treated group compared to control, BFPD (500 mg/kg) + HgCl_2_ and BFPD (1000 mg/kg) + HgCl_2_ groups. Similarly, the silymarin + HgCl_2_-treated group decreased in the number of hepatocytes when compared to the control (Fig. 8B).

### Molecular assessments

Molecular assessment of liver GPx gene expression revealed a significant down regulation in HgCl_2_-treated group when compared to both the control and silymarin + HgCl_2_-treated groups (Fig. 8C).

### Biochemical assessments

**Fig. 9:**
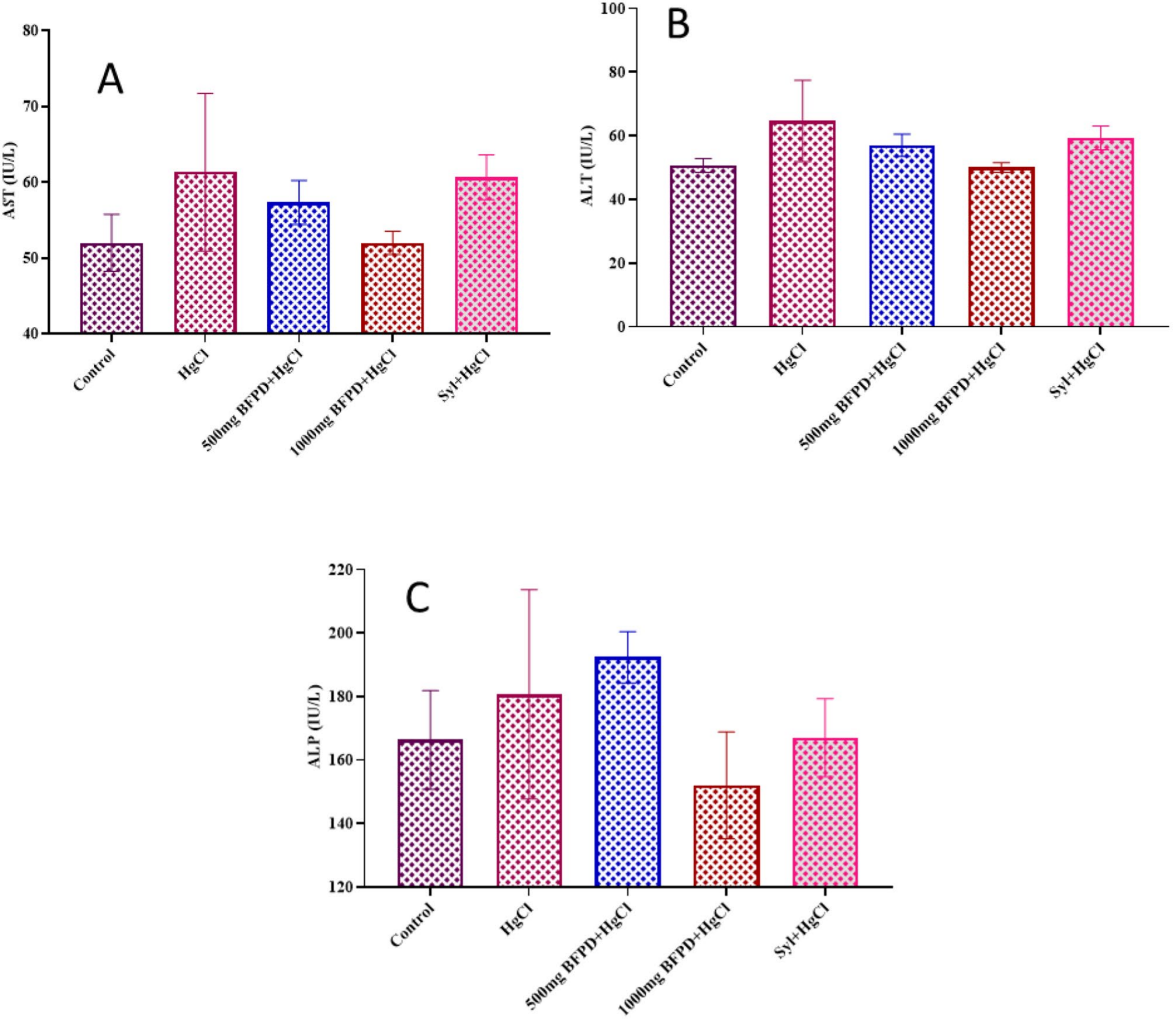
**(A)** Effect of BFPD on liver serum enzyme (aspartate transaminase) activity of Wistar rats. *n* = 5, mean ± SEM, one way ANOVA, *p* < 0.05 when compared across the group. HgCl_2_ = Mercury chloride (5 mg/kg), BFPD = *n*-Butanol fraction of *Phoenix dactylifera* (500 mg/kg; 1000 mg/kg), Syl = silymarin (100 mg/kg). **(B)** Effect of BFPD **on** liver serum enzyme (alanine transaminase) activity of Wistar rats. *n* = 5, mean ± SEM, one way ANOVA, *p* < 0.05 when compared across the group. HgCl_2_ = Mercury chloride (5 mg/kg), BFPD = *n*-Butanol fraction of *Phoenix dactylifera* (500 mg/kg; 1000 mg/kg), Syl = silymarin (100 mg/kg). **(C)** Effect of BFPD on liver serum (alkaline phosphatase) activity of Wistar rats. *n* = 5, mean ± SEM, one way ANOVA, *p* < 0.05 when compared across the group. HgCl_2_ = Mercury chloride (5 mg/kg), BFPD = *n*-Butanol fraction of *Phoenix dactylifera* (500 mg/kg; 1000 mg/kg), Syl = silymarin (100 mg/kg)

Biochemical assessments of liver serum enzymes AST, ALT, and ALP; liver serum proteins (AB), GB, and TP; and oxidative stress biomarkers (Superoxide dismutase (SOD), Catalase (CAT), GPx, and Malondialdehyde (MDA)) revealed the following:

Assessment of liver serum enzymes of Wistar rats revealed slight (*p* > 0.05) elevation in the activities of AST, ALT, and ALP in the treated groups except for BFPD (1000 mg/kg) + HgCl_2_ compared to control (Fig. 9A– C).

**Fig. 10:**
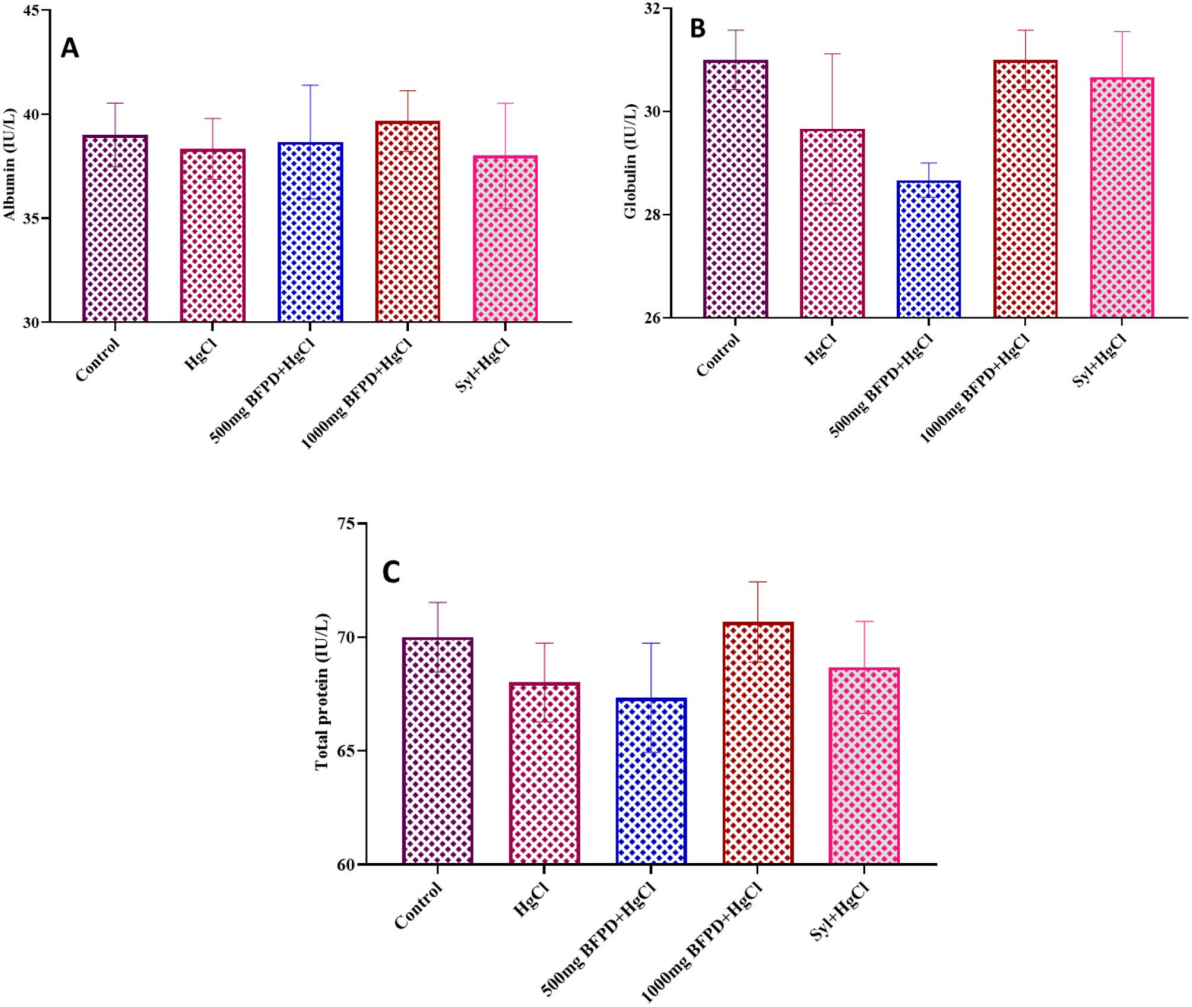
**(A)** Effect of BFPD on liver serum albumin levels of Wistar rats. *n* = 5, mean ± SEM, one way ANOVA, *p* > 0.05 when compared across the group. HgCl_2_ = Mercury chloride (5 mg/kg), BFPD = *n*-Butanol fraction of *Phoenix dactylifera* (500 mg/kg; 1000 mg/kg), Syl = silymarin (100 mg/kg). **(B)** Effect of BFPD on liver serum globulin levels of Wistar rats. *n* = 5, mean ± SEM, one way ANOVA, *p* > 0.05 when compared across the group. HgCl_2_ = Mercury chloride (5 mg/kg), BFPD = *n*-Butanol fraction of *Phoenix dactylifera* (500 mg/kg; 1000 mg/kg), Syl = silymarin (100 mg/kg). **(C)** Effect of BFPD on liver serum total protein levels of Wistar rats. *n* = 5, mean ± SEM, one way ANOVA, *p* > 0.05 when compared across the group. HgCl_2_ = Mercury chloride (5 mg/kg), BFPD = *n*-Butanol fraction of *Phoenix dactylifera* (500 mg/kg; 1000 mg/kg), Syl = silymarin (100 mg/kg)

Liver serum proteins of Wistar rats revealed slight (*p* > 0.05) decreased levels of AB, GB and TP in the treated groups except BFPD (1000 mg/kg) + HgCl_2_ relative to the control (Fig. 10A, B & C).

**Fig. 11:**
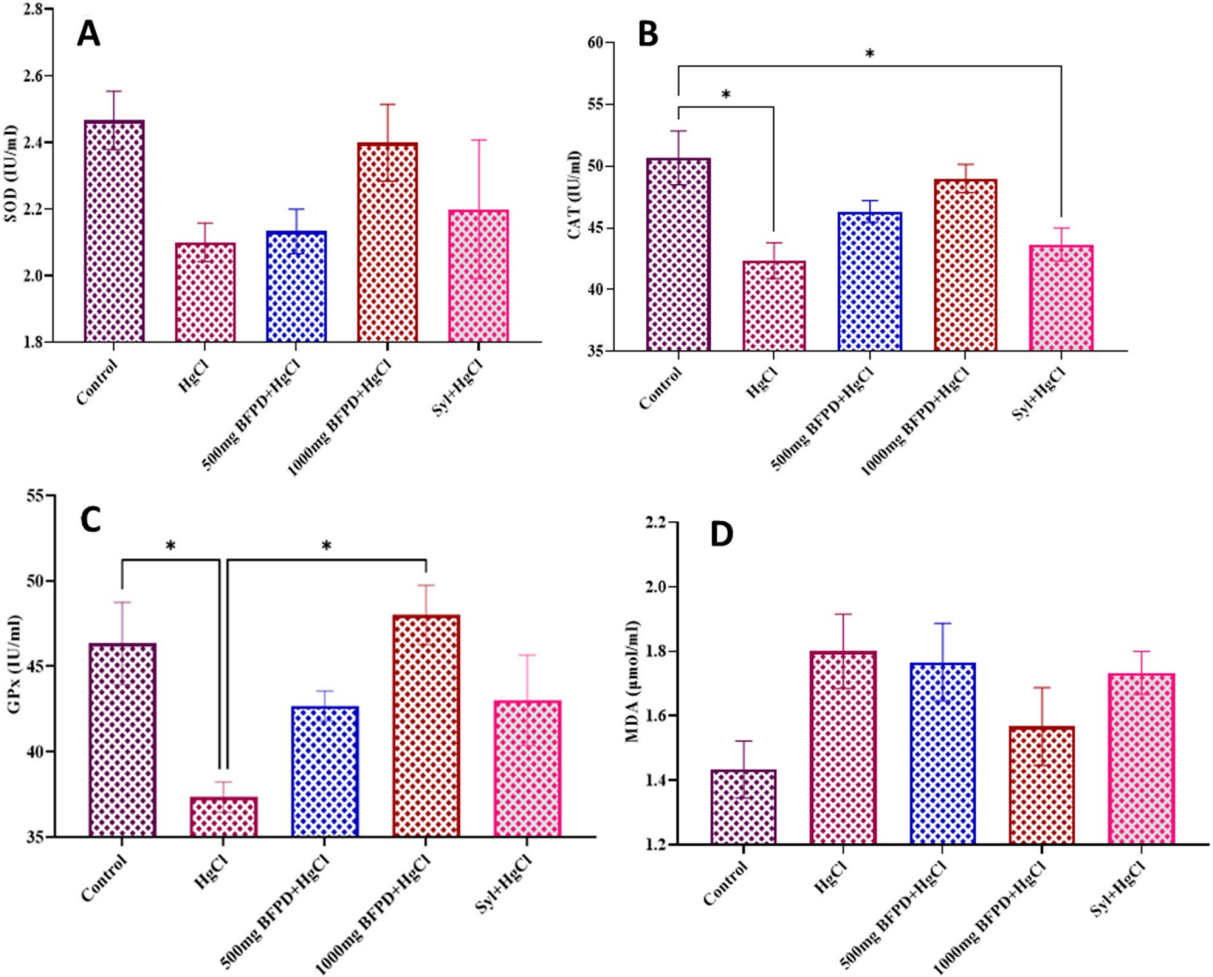
**(A)** Effect of BFPD on superoxide dismutase concentration of Wistar rats. *n* = 5, mean ± SEM, one way ANOVA, *p* < 0.05 when compared across the group. HgCl_2_ = Mercury chloride (5 mg/kg), BFPD = *n*-Butanol fraction of *Phoenix dactylifera* (500 mg/kg; 1000 mg/kg), Syl = silymarin (100 mg/kg). **(B)** Effect of BFPD on catalase concentration of Wistar rats. *n* = 5, mean ± SEM, one way ANOVA, *=*p* < 0.05 when control was compared to the HgCl_2_ group and when control was compared to the Silymarin group. **(C)** Effect of BFPD on glutathione peroxidase concentration of Wistar rats. *n* = 5, mean ± SEM, one way ANOVA, *=*p* < 0.05 when control was compared to HgCl_2_ group, and when Hgcl_2_ was compared to 1000 mg BFPD group. HgCl_2_ = Mercury chloride (5 mg/kg), BFPD = *n*-Butanol fraction of *Phoenix dactylifera* (500 mg/kg; 1000 mg/kg), Syl = silymarin (100 mg/kg). HgCl_2_ = Mercury chloride (5 mg/kg), BFPD = *n*-Butanol fraction of *Phoenix dactylifera* (500 mg/kg; 1000 mg/kg), Syl = silymarin (100 mg/kg). **(D)** Effect of BFPD on malondialdehyde concentration of Wistar rats. *n* = 5, mean ± SEM, one-way ANOVA, *p* < 0.05 when compared across the group. HgCl_2_ = Mercury chloride (5 mg/kg), BFPD = *n*-Butanol fraction of *Phoenix dactylifera* (500 mg/kg; 1000 mg/kg), Syl = silymarin (100 mg/kg)

Assessment of oxidative stress biomarkers associated with serum of Wistar rats revealed decreased (*p* > 0.05) activities of SOD and CAT in all the treated groups relative to the control (Fig. 11A and B). GPx activity decreased (*p* > 0.05) in BFPD (500 mg/kg) + HgCl_2_ and silymarin + HgCl_2−_treated groups with significant decrease in HgCl_2−_treated group when compared to the control, while BFPD (1000 mg/kg) + HgCl_2_ increased (*p* < 0.05) as compared to HgCl_2−_treated group. (Fig. 11C). Relative to the control, MDA levels increased in all treated groups (Fig. 11D).

## Discussion

Mercury exposure can overwhelm the liver making it challenging to perform their biological and physiological functions optimally [22]. HgCl_2_ has also been reported to be a potent hepatotoxic agent [23, 24]. The liver plays a critical role in the elimination and detoxification of xenobiotic substances [25]. Hepatic injury can be caused by hepatotoxic substances which are triggered by lipid peroxidation as well as several oxidative damages [26].

*Phoenix dactylifera* is reported for various pharmacological activities including anti- inflammatory, hepatoprotective, antioxidant and many more [27, 28]. It acts as an antioxidant checkmating free radical-induced tissue damage, inhibits lipid peroxidation, and alters drug-induced histopathological changes [13, 29]. In this study, Phytochemical analysis of BFPD revealed the presence of flavonoids, saponins, and tannins among others which have been reported to exert hepatoprotective actions [30–32] in animals and cell culture models. Silymarin has been used for centuries to treat liver disorders [31]. The antioxidant property of silymarin is well established and used in the evaluation of potential hepatoprotective agents [13, 31].

Physically observed behavioral patterns are important pointers for animal well-being, especially rodents [33]. Reduced physical activity including sluggishness and aggression observed in the mercury-treated group when compared to the control group could be a result of toxicity resulting in loss of appetite and improper assimilation of food. This finding is in line with the report of Ansar and AlGhosoon [34], and Amber et al. [17] who observed rats exposed to mercuric chloride triggered physiological changes including reduced agility and feeding activity. Agbon et al. [14] reported reduced physical activity following treatment with mercuric chloride in rats.

Absolute body weight changes have been reported as an indicator of the health status of animals [35]. A lower absolute body weight change observed in the HgCl_2_-treated group when compared to the control could be associated with HgCl_2_-induced toxicity. This accords with Thomas et al. [36], and Jadhav et al. [37] who observed that the body weight of rats treated with HgCl_2_ decreased significantly when compared to the controls.

In this study, there were no significant differences in relative liver body weight ratio (organosomatic index) when compared to the control. This implies that the treatment probably did not affect relative liver body weight ratio values. These findings agree with Ajibade et al. [38] who reported no significant difference in organ‑body weight ratio when exposed to HgCl_2_. Similarly, Amber et al. [17] observed that oral administration of HgCl_2_ and plant extract did not affect the organ-body weight ratio in Wistar rats.

The first organ to encounter ingested nutrients, drugs and environmental toxins is the liver, as such, acute or chronic exposure to heavy metals such as mercury can cause severe injury to the liver and lead to a decline in its function [39]. According to Wargovich et al. [40], and Kumari and Chand [41], mercury chloride is one of the most toxic forms of mercury because it easily forms organomercuric complexes with proteins in a biological system. HgCl_2_ has also been reported to be a potent hepatotoxic agent [23, 42]. The liver is the major site of drug metabolism including mercury resulting in severe alterations to the structural and functional unit of this organ [43]. The liver plays a critical role in the elimination and detoxification of xenobiotic substances [24]. Hepatic injury can be caused by hepatotoxic substances which are triggered by lipid peroxidation as well as several oxidative damages [25].

This study observed histoarchitectural distortion of the liver in the HgCl_2_-treated group such as hepatocellular vacuolation, congestion of the central vein, and sinusoidal dilatation which could be attributed to HgCl_2_-induced hepatotoxicity. This is in concordance with Adams et al. [44], Ibegbu et al. [45], and Goudarzi et al. [46] who reported that rats treated with HgCl_2_ revealed congested central vein, dilated sinusoids, and necrotic hepatocytes. Pyknotic changes are the hallmark of cell injury [47]. In this study, hepatocellular pyknotic nuclei suggestive of hepatotoxicity were observed. This agrees with the report revealing darkly stained nuclei associated with pyknosis in rats treated with mercury [14, 44, 47]. Observed hepatocellular vacuolation in this study agrees with the report of Singh et al. [48], Ibegbu et al. [45], and Goudarzi et al. [46] who demonstrated histoarchitectural distortions as cytoplasmic vacuolation in hepatocytes exposed to mercury-induced oxidative stress.

*Phoenix dactylifera*’s high therapeutic effects have increased its use, encouraged by the growing consumer concern for health. Silymarin is a polyphenolic compound extracted from *Silibum marianum* and *Cynara cardunculus* seeds and fruits. It acts as an antioxidant checkmating free radical-induced tissue damage, inhibits lipid peroxidation, and alters drug-induced histopathological changes [13, 28].

In this study, groups administered BFPD and silymarin both followed by HgCl_2_ showed preserved histoarchitecture of the liver similar to that of the control group. This suggests that BFPD and silymarin both counteract HgCl_2−_induced hepatotoxicity in rats. This is in line with the works of Goudarzi et al. [49] who reported that plant extract alleviates oxidative stress and inflammation in rats and Baradaran et al. [50] who reported on the hepatoprotective effect of silymarin. The hepatoprotective effect of BFPD could be attributed to its antioxidant property of constituent phytochemicals such as flavonoids; antioxidants have been reported to abate oxidative stress-associated pathologies directly, by scavenging reactive oxygen species (ROS) [51].

The normal healthy liver hepatocytes store large quantities of glucose (as glycogen) after a meal and release it when fasting [52, 53]. The presence of glycogen leads to the staining of hepatocytes intensely purple with PAS stain [54]. Liver glycogen analysis in this study demonstrated reduced PAS staining intensity in the HgCl_2_-treated group which indicates glycogen depletion and cytoarchitectural alteration of the liver tissue as a result of HgCl_2_ toxicity. This accords with the study of Mohamed et al. [55] who observed weak PAS reaction with HgCl_2_ treatment in experimental rat models. Remarkable PAS staining intensity observed in BFPD (1000 mg/kg) followed by HgCl_2_ as compared to the HgCl_2_-treated group suggests glycogen moiety preservation and hence, hepatoprotection against HgCl2-induced cytoarchitectural changes. Silymarin treated group revealed PAS staining intensity similar to the control. This concurs with Boyd et al. [56], who reported that PAS staining intensity increases with abundant glycogen and decreases with glycogen depletion.

Reticular fibers are a special type of connective tissue composed of collagen III. These fibers are arranged delicately to form a mesh-like network that provides support to the hepatocytes and sinusoids [57]. Since reticulin provides stromal support for the liver parenchyma, the reticulin stain (that is, MT) provides important information about the architectural integrity of the liver. As regards this study, MT stain revealed no distinct difference in reticulin framework across the treated groups relative to the control. This result agrees with Singhi [58] and Yasir [59] who reported that reticulin loss was not associated with the degree of inflammation or with the presence or absence of alteration in cell change.

An established mechanism of HgCl_2_-induced liver injury involves free radical-mediated damage, reactive oxygen species (ROS) production, and ultimately cell death [60]. Reactive oxygen species are predominantly generated in mitochondria and play a key role in apoptosis [61]. The expressions of Bcl-2 protein play a pivotal role in the regulation of apoptotic cell death and inhibit the production of free radicals and oxidative stress-induced cell death [21]. Apoptosis signaling pathways involve altering the Bcl-2 (antiapoptotic) and BAX (proapoptotic) protein ratio [16].

The current study demonstrated that HgCl_2_-treated group stained less immunopositively for Bcl-2 as regards the control, indicating the suppression of Bcl-2 and expression of BAX proteins thereby propagating apoptosis in liver cells. A similar study has been reported by Yang [60], and Roshankhah [62] indicating that HgCl_2_ induced apoptosis by increasing the expression of BAX in rat liver tissue. One of the important apoptotic pathways is the mitochondrial (intrinsic) pathway characterized by increased cytochrome-C levels in the cytoplasm due to the impaired Bcl-2/Bax balance. The increased level of cytochrome-C results in caspase-3 activation thereby promoting apoptosis via the intrinsic pathway [63, 64]. It has been reported that mercury increases mitochondrial membrane permeability followed by the mitochondrial membrane potential reduction [65].

However, the protective effect of BFPD and silymarin was revealed in rats pre-treated with BFPD and silymarin followed by HgCl_2_ where staining was more immuno-positive for Bcl-2 by retaining Bcl-2 expressions in the liver cells, this was similar to the control. Roshankhah [62] reported that extracts of *P. dactylifera* upregulate Bcl-2 and downregulate apoptotic factors such as BAX. Additionally, Caglayana [16] reported that plant extracts with antioxidant activity suppress proapoptotic proteins.

Stereological methods were used to estimate the number of hepatocytes in the liver. In this study, the total number of normal hepatocytes was remarkably reduced in the group exposed to HgCl_2_ as compared to the control. This occurrence is a result of degeneration and death of cells caused by HgCl_2_ toxicity. These findings are in line with Yahyazedeh [66] who observed that exposure to mercury (Hg) vapor caused a reduction in the total number of hepatocytes.

On the other hand, BFPD and silymarin groups followed by HgCl_2_ administration revealed an appreciable increase in the number of hepatocytes compared to the HgCl_2_-treated group. Thus, suggesting the preservation of microscopic features. This coincides with the report that extracts of *P. dactylifera* could serve as a potent antioxidant that effectively attenuates the adverse effects of HgCl_2_ [62].

In the present study, we performed molecular assessments to further support the histopathological, immunological, stereological, and biochemical evidence obtained. Our analysis revealed that the expression of GPx antioxidant gene was remarkably downregulated in the hepatic tissues by HgCl_2_ exposure in a similar pattern to GPx enzyme activity. This suggests that the HgCl_2_ accumulation in the biological tissues overwhelmed the antioxidant mechanism and caused antioxidant levels to drop.

Contrarily, pre-treatment with BFPD and silymarin showed an upregulation in the expression of the GPx gene in the liver tissue. This result is parallel to Ramadan [20] and Othman [21] who relayed that antioxidant genes were down-regulated by mercury exposure and upregulated by administration of plant extracts with antioxidant potential.

Elevation of serum AST, ALT, and ALP activities are important markers of hepatocellular damage and liver diseases [67, 68]. When the liver suffers an injury, cytoplasmic enzymatic biomarkers such as AST, ALT, and ALP leak from the liver into the systemic circulation [69], resulting in increased levels of these enzymes in the serum. Observed elevated activities of AST, ALT, and ALP in the HgCl_2_–treated group compared to control could be attributed to hepatocellular injury and damage to the plasma membrane resulting in the leakage of such enzymes. The elevation in liver enzymes is well supported by Ajibade et al. [38], Nabil et al. [70] and Abdelghani et al. [71] who observed a remarkable increase in the activities of liver enzymes due to exposure to HgCl_2_, a known trigger of significant liver injury. On the other hand, enzymatic (AST, ALT, and ALP) activities following BFPD (1000 mg/kg)-administration tilted towards normal relative to the HgCl_2_–treated group. This suggests hepatoprotection. This finding could be attributed to the membrane-stabilizing activity of phytochemical constituents of BFPD that prevents the leakage of intracellular enzymes.

Decreased levels of serum liver proteins (AB, GB, and TP) point to the possibility of liver injury. Liver proteins are synthesized by the liver, but in the presence of toxicity, the process of protein formation is disrupted which leads to low serum protein concentration [72]. This study observed decreased serum levels of liver proteins in the HgCl_2_-treated group. These observations accord with Ekam and Udosen [73], and Lala [74] who related decreased serum liver proteins to abnormal liver functions. Other treated groups did not reveal any significant change in the serum proteins as compared to the control group and HgCl_2_-treated group. Abd Elghani [71] reported similar findings in his study.

In this study, relative to antioxidant enzyme activity, it was noted that HgCl_2_ diminished the activities of enzymatic antioxidants such as SOD and CAT (with a remarkable decline in CAT activity) in liver tissues. HgCl_2_ also diminished the activity of enzymatic antioxidant GPx (with a remarked decline in activity), whereas oxidative stress biomarkers (MDA contents) which is the end product of lipid peroxidation increased compared to the control group. These findings are in agreement with the reports of Nabil et al. [70], and Manju and Jagadeesan [75], who implied that the high accumulation of toxic metabolites in the system resulted from HgCl_2_-induced oxidative stress. Goudarzi et al. [49] equally documented the reduction of antioxidant enzymes as a result of exposure to environmental toxins.

Conversely, co-administration of BFPD + HgCl_2_ and silymarin + HgCl_2_ modulated SOD, CAT and MDA activities slightly toward normal. GPx activity was remarkably elevated by BFPD (1000 mg/kg) + HgCl_2_. This indicates that the ameliorative potential of BFPD could be associated with antioxidant activity aimed against HgCl_2_-triggered toxicity owing to oxidative stress. These observations concur with Ahmed et al. [76], and Goudarzi et al. [49] who stated that plant extracts inhibit oxidative stress by decreasing lipid peroxidation in drug-induced toxicity. Agbon et al. [14] related the protective potential of *P. dactylifera* to its antioxidant properties.

Phytonutrients with antioxidant activities including polyphenolics; especially flavonoids, have been associated with hepatorenal protective properties in experimental animal models. Flavonoids are beneficial phytochemical constituents of *P. dactylifera* reported to play a critical role as free radical scavengers or antioxidants in biological systems [77, 78]. The most emphasized antioxidant property of flavonoids is derived from their ability to directly scavenge the reactive oxygen species; flavonoids can chelate free radicals immediately by donating a hydrogen atom or by single-electron transfer or binding to metal ions in the human body to prevent them from being accessible for oxidation [79, 80].

## Conclusions

From the results obtained, it was concluded that *n-*butanol fraction of *Phoenix dactylifera L*. possesses the potential to protect against HgCl_2_-induced alterations in the liver of Wistar rats. *n-*butanol fraction of *Phoenix dactylifera L*. is dose dependent as 1000 mg/kg proved to be more effective. The hepatoprotective efficacy of *n-*butanol fraction of *Phoenix dactylifera L*. is comparable to, or in some instances, more efficacious than the reference drug (silymarin). Hepato- protection could be attributed to the constituent antioxidant properties especially flavonoids in BFPD.

Therefore, *n-*butanol fraction of *Phoenix dactylifera L*. may be a novel candidate for treating and managing liver-induced inorganic mercury toxicity.

## Data Availability

Not applicable.

## Abbreviations

AB: Albumin
ABU: Ahmadu Bello University
AST: Aspartate Aminotransferase
ALT: Alanine Transaminase
ALP: Alkaline Phosphatase
BCL2: B Cell Lymphoma 2
BFPD: *n* butanol Fraction of *Phoenix dactylifera*
CAT: Catalase
GB: Globulin
GPx: Glutathione Peroxidase
H&E: Haematoxylin and Eosin
MDA: Malondialdehyde
Mg/kg: milligram per kilogram body weight
MT: Mason Trichome
PAS: Periodic Acid Schiff
ROI: Region of Interest
ROS: Reactive Oxygen Species
SOD: Superoxide Dismutase
TP: Total Proteins
